# Proactive Therapeutic Drug Monitoring of Dalbavancin in the Long-Term Treatment of Chronic Infections: A Narrative Review

**DOI:** 10.3390/antibiotics15030253

**Published:** 2026-03-01

**Authors:** Dario Cattaneo, Jessica Cusato

**Affiliations:** 1 Department of Biomedical Sciences, Humanitas University, 20072 Pieve Emanuele, Italy; 2 Clinical Analysis Laboratory, IRCCS Humanitas Research Hospital, 20089 Rozzano, Italy; 3 Department of Medical Sciences, University of Turin, 10126 Turin, Italy; jessica.cusato@unito.it

**Keywords:** dalbavancin, therapeutic drug monitoring, pharmacokinetics, pharmacodynamics, chronic infections

## Abstract

Dalbavancin is a long-acting lipoglycopeptide antibiotic increasingly off-label used for the management of complex and chronic Gram-positive infections, including osteoarticular, prosthetic, and cardiovascular device-related infections. While its prolonged half-life enables infrequent dosing, marked inter-individual pharmacokinetic variability has been documented during extended treatment courses, potentially resulting in suboptimal exposure. This narrative review explores the role of proactive therapeutic drug monitoring (TDM) as a strategy to individualize dalbavancin dosing in patients requiring long-term therapy. We summarized current evidence on pharmacokinetic determinants of dalbavancin exposure, including renal function, body weight, and hypoalbuminemia, and discussed proposed pharmacokinetic/pharmacodynamic targets to support TDM implementation. Available analytical methods for dalbavancin quantification and clinical experiences with TDM-guided dosing are reviewed, highlighting their impact on optimizing injection timing and maintaining adequate drug concentrations over prolonged periods. In addition, emerging model-informed precision dosing approaches, such as Bayesian forecasting and machine learning-based tools, are discussed as promising strategies to further refine exposure prediction and re-dosing decisions. Overall, proactive TDM represents a valuable tool for optimizing dalbavancin therapy in chronic infections, although prospective multicenter studies are needed to validate target thresholds and standardized implementation strategies.

## 1. Introduction

Dalbavancin is a long-acting parenteral lipoglycopeptide antibiotic that inhibits bacterial growth primarily by targeting the terminal D-alanyl-D-alanine residues of peptidoglycan precursors, thereby disrupting the function of transpeptidase and transglycosylase enzymes. Approximately a decade ago, it received regulatory approval from both the U.S. Food and Drug Administration (FDA) and the European Medicines Agency (EMA) for the management of acute bacterial skin and skin structure infections (ABSSSI) [1,2,3,4,5].

Dalbavancin exhibits distinctive pharmacokinetic and pharmacodynamic (PK/PD) properties. Its prolonged half-life allows the treatment of ABSSSI with a single 1500 mg dose (a two-dose regimen consisting of 1000 mg followed by 500 mg one week later may also be considered) [3,6]. The extended terminal half-life (9–14 days) is partially attributable to its high plasma protein binding, particularly to albumin (approximately 93%) [3].

Dalbavancin is active against a broad range of Gram-positive pathogens, including Staphylococcus aureus with intermediate susceptibility to vancomycin, multidrug-resistant coagulase-negative staphylococci, methicillin-resistant Staphylococcus aureus (MRSA) and vancomycin-resistant enterococci [1,2,3,4,5]. Growing evidence supports the prolonged use of dalbavancin in the management of complex chronic infections [7,8]. However, dosing strategies vary considerably across studies, and clear recommendations for extended dalbavancin regimens remain limited. Recently, an expert review panel proposed the use of therapeutic drug monitoring (TDM) as a strategy to optimize dosing in patients requiring dalbavancin therapy for longer than six weeks [9].

In this narrative review, we evaluated the potential role of proactive TDM, defined as the measurement of drug concentrations at predefined time points, irrespective of clinical symptoms or treatment failure, with subsequent dose adjustment to maintain concentrations within a target therapeutic range. This is considered a possible tool for optimizing the timing of dalbavancin administration in patients with chronic infections.

## 2. Search Strategy

A MEDLINE PubMed search was conducted for articles published between January 2000 and December 2025, matching the terms “pharmacokinetics” or “therapeutic drug monitoring” with “chronic” or “prolonged” and with “dalbavancin”. Only articles published in English were included in this narrative review. Additional studies were identified from the reference lists of retrieved articles.

## 3. The Rationale for Prolonged/Chronic Treatments with Dalbavancin

Dalbavancin, owing to its potent activity against Gram-positive cocci, extended half-life, and favorable safety profile, represents a promising alternative to daily intravenous or oral antibiotics for the management of infections requiring prolonged or chronic treatments [7,8]. Dalbavancin is frequently used off-label for the treatment of bone and joint infections, prosthetic joint infections, infective endocarditis, vascular graft infections, and other Gram-positive infections associated with biofilm formation [10,11,12,13]. These infections often necessitate weeks to months of therapy, sometimes combined with surgical debridement, or long-term suppressive treatment when source control is not feasible. Patients who fail initial therapy or are considered unsuitable for surgery because of frailty or significant comorbidities are the most frequent candidates for long-term suppressive treatment. Such regimens commonly rely on beta-lactams or tetracyclines and are often burdened by a high incidence of adverse events [10,11,12,13]. Management is particularly difficult in infections caused by Staphylococcus aureus and other staphylococcal or streptococcal species, owing to biofilm formation and poor antibiotic penetration into bone and joint tissues [14,15]. In this context, dalbavancin exhibits favorable distribution within these compartments [16,17], supporting its potential role in the management of complex infections, although these uses are not yet covered by current regulatory approvals.

## 4. Clinical Factors Affecting Dalbavancin Pharmacokinetics

Some clinical factors may influence dalbavancin PK, particularly during prolonged or repeated dosing regimens. Renal function represents the primary determinant of dalbavancin clearance, with renal elimination accounting for up to approximately 45% of total drug elimination [3]. While no dose adjustment is required in patients with mild or moderate renal impairment, reduced clearance and increased exposure have been observed in patients with severe renal dysfunction, for whom dose reduction is recommended. According to the drug monograph, a reduced dalbavancin dose of 1000 mg is recommended for patients with creatinine clearance (CLCR) <30 mL/min, whereas patients with augmented renal clearance (ARC) may be at risk of underexposure, although dedicated studies are lacking. To date, only a single case reporting an atypical pharmacokinetic profile in a child with ARC has been described [18]. In this patient, ARC was associated with a marked reduction in the half-life of dalbavancin (2.4 days).

These recommendations apply to the approved single-dose regimen for acute bacterial skin and skin structure infections. A population PK/PD analysis by Cojutti et al. evaluated dalbavancin for long-term treatment across different degrees of renal function using 1000-subject Monte Carlo simulations [19]. In addition to approved regimens, two intensified schedules were assessed. Approved dosing ensured optimal probability of target attainment (PTA) for 2–3 weeks, depending on renal function, while double-dosing one week apart extended optimal PTA up to 4–6 weeks. Despite renal stratification, substantial inter-individual variability in dalbavancin exposure persisted within each CLCR class. Consequently, the authors proposed proactive, individualized TDM strategies to anticipate declines in drug concentrations below optimal exposure thresholds [19].

Hepatic impairment has a negligible impact on dalbavancin PK, as the drug undergoes minimal hepatic metabolism; accordingly, no dose adjustment is recommended in patients with hepatic dysfunction [3]. Dalbavancin is neither a substrate, inhibitor, nor inducer of cytochrome P450 enzymes, and clinically relevant drug–drug interactions (DDIs) are therefore unlikely. Similarly, no clinically meaningful differences in PK have been reported with respect to age or sex after adjustment for renal function [3].

Dalbavancin is highly bound to plasma proteins, predominantly albumin (93%), and hypoalbuminemia (defined as serum albumin <20 g/L and frequently observed in critically ill patients and in those with chronic inflammatory conditions) may increase the unbound fraction of the drug, potentially affecting its distribution and clearance. In a prior study, serum albumin levels showed a significant association with the timing of dalbavancin administration in univariate analyses [20]; however, this factor was not included in the multivariable model, likely because none of the patients enrolled exhibited severe hypoalbuminemia. In contrast, a previously published case series described a patient with markedly accelerated dalbavancin clearance who had profound hypoalbuminemia (14 g/L) and was receiving continuous renal replacement therapy [21]. On this basis, it has been hypothesized that severe hypoalbuminemia may increase the free (unbound) fraction of dalbavancin, thereby enhancing renal elimination, particularly in patients undergoing dialysis. Supporting this hypothesis, Carrothers et al. recently reported a significant correlation between serum albumin concentrations and dalbavancin clearance in a population PK model [22]. The identification of optimal PK targets for dalbavancin in patients with severe hypoalbuminemia represents one of the key objectives for future research in this field.

Emerging but consistent evidence suggests that extreme body weight may influence dalbavancin volume of distribution and systemic exposure, contributing to interindividual PK variability. In 2023, Hervochon et al. reported significantly lower dalbavancin concentrations in patients weighing >75 kg compared with those of lower body weight [23]. These results were supported by Cattaneo et al., who, for the first time, reported a progressive and significant shortening of the dosing interval alongside a corresponding increase in the volume of distribution in patients across normal-weight, overweight, and obese categories [20]. Similarly, Baiardi et al., using a multidose population PK model, demonstrated that two 1500 mg dalbavancin doses administered seven days apart achieved an optimal PTA (>90% for 100% fT >4 × MIC) for up to 5, 4, and 3 weeks in patients weighing 40–80 kg, 80–120 kg, and 120–200 kg, respectively [24].

In patients with obesity, the volume of distribution is often increased due to expanded fat and lean body mass. Consequently, overweight and obese individuals may be at risk of suboptimal dalbavancin exposure when receiving the standard 1500 mg dose. This issue is likely of limited clinical significance when dalbavancin is administered as a single dose for the treatment of ABSSSI. Supporting this, a subgroup analysis of pooled Phase 3 clinical trials showed that single-dose dalbavancin maintained clinical efficacy in patients with obesity or diabetes [25]. However, a further sub-analysis from the trial by Dunne et al. indicated a non-significant trend toward lower clinical success rates in patients with body mass indices of 35–40 kg/m^2^ and ≥40 kg/m^2^ compared with other treatment groups [26].

Obesity may pose a greater challenge when dalbavancin is administered as prolonged or chronic therapy. In this context, Ritchie et al. recently reported clinical failure for dalbavancin treatment in a patient with severe obesity and MRSA bacteraemia, complicated by intravenous drug use [27]. Despite partial dalbavancin treatment, the patient developed vertebral osteomyelitis and discitis with an associated epidural phlegmon, likely representing complications of persistent MRSA bacteraemia.

**Figure 1:**
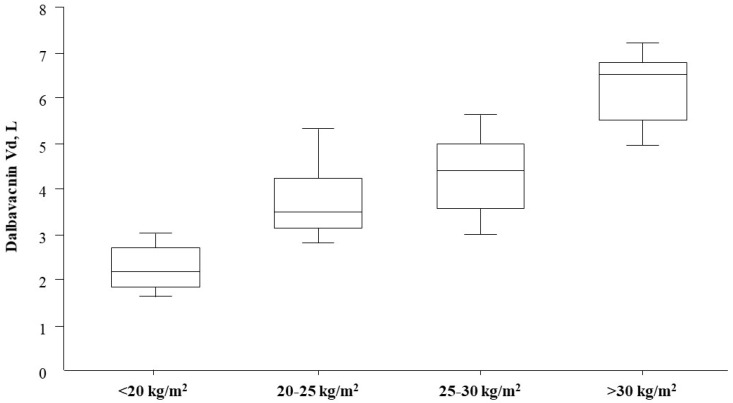
Box plot of dalbavancin volumes of distribution clustered according to BMI (the boxes/lines/whiskers represent the 5th, 25th, 50th, 75th and 95th percentile values).

Notably, we recently demonstrated that severe underweight may also substantially alter dalbavancin PK [28]. In this instance, the severely underweight patient displayed a substantially lower volume of distribution compared with normal-weight, overweight, and obese individuals. As shown in Figure 1, a linear relationship has been observed between Vd and BMI. As a result, severely underweight patients could theoretically be at risk of drug overexposure when receiving the standard 1500 mg dalbavancin dose, especially during prolonged or chronic treatment, although no plasma concentration threshold for dalbavancin-related toxicity has been established to date. Indeed, in the absence of established plasma concentration thresholds for dalbavancin-related toxicity, proactive TDM may primarily serve to support rational resource use and avoid unnecessary drug accumulation, rather than preventing known toxic effects.

Collectively, these observations support the consideration of individualized, TDM-guided dosing strategies, particularly in patients requiring long-term dalbavancin therapy and in those with extreme body weight, altered renal function, or significant hypoalbuminemia.

## 5. Definition of Therapeutic Targets for the TDM of Dalbavancin

Before implementing TDM, it is essential to define the drug therapeutic concentration window. To date, no dalbavancin exposure thresholds associated with dose-dependent toxicity have been identified. In contrast, more evidence is available regarding lower concentration limits, below which the risk of therapeutic failure may increase. The first approach, described by Cojutti et al. in 2021, involves several key considerations: (a) achieving a 24 h area under the concentration-time curve for the free (unbound) fraction of dalbavancin relative to the minimum inhibitory concentration (fAUC_(24h)_/MIC) above 111, defined as the optimal PK/PD target against Staphylococcus aureus; (b) determining the fAUC_(24h)_ required to reach this target for Staphylococcus aureus strains with MICs up to the EUCAST clinical breakpoint for dalbavancin susceptibility (0.125 mg/L); (c) accounting for dalbavancin high plasma protein binding (93%); and (d) considering the established correlation between dalbavancin C_min_ and AUC [29]. Based on these parameters, the target Cmin was calculated to be 8.0 mg/L. Alternatively, a conservative PK/PD target can be established a priori by setting it at four times the EUCAST breakpoint for staphylococcal species (4 × 0.125 mg/L = 0.5 mg/L for the free fraction), and then adjusting for dalbavancin plasma protein binding (93%), resulting in a total C_min_ threshold of 7.2 mg/L. The advantage of this approach is that it does not rely on knowledge of the C_min_-AUC relationship, which may not always be available.

## 6. Analytical Methods for the Quantification of Dalbavancin in Biological Matrices

Different analytical methods for the quantification of dalbavancin have been reported in the literature, with the majority being developed and validated for serum or plasma samples [30,31,32,33,34,35,36]. As summarized in Table 1, all of these methods are chromatographic and employ UV, diode-array, or mass spectrometric detection. The main differences among the methods lie in their linearity ranges and limits of quantification (LOQ). Notably, some methods are highly sensitive, reporting LOQ values below 1 mg/L. These differences may become clinically relevant when dalbavancin is used to treat pathogens with very low MICs (e.g., <0.0625 mg/L). In such scenarios, methods with higher LOQs may not allow accurate assessment of PK/PD targets. Conversely, other methods report upper limits of quantification of 50, 100, or 200 mg/L [32,34,35]. This may pose challenges for the application of TDM based on the evaluation of peak dalbavancin concentrations (Cmax), which are typically higher than 200 mg/L (see next chapters). Similarly, a method with a LOQ of 12.5 mg/L may be inadequate for accurate proactive TDM at the lower end of the therapeutic window [31]. Although this limitation can be addressed by sample dilution, this approach increases sample processing time and laboratory workload.

## 7. Preliminary Experience with the TDM of Dalbavancin

Corona et al. (2020) were the first to highlight the potential role of therapeutic drug monitoring (TDM) in optimizing dalbavancin therapy [21]. Their study, which involved three critically ill patients with necrotizing fasciitis, demonstrated substantial inter-individual variability in dalbavancin exposure, with one patient showing a six-fold higher drug clearance than the others.

A few months later, Cojutti et al. reported a proof-of-concept study demonstrating the usefulness of TDM in estimating the duration of optimal dalbavancin target attainment in patients with staphylococcal osteoarticular infections, as already discussed in the chapter addressing the definition of therapeutic ranges [29]. Subsequently, the same group published a series of studies further reinforcing the clinical relevance of TDM for optimizing dalbavancin dosing, particularly in terms of injection timing, across different clinical settings [37,38,39,40]. The first of these studies focused on the off-label use of dalbavancin as sequential therapy for MRSA spondylodiscitis [37]. Authors demonstrated that dalbavancin represents a safe and effective sequential treatment option for patients with severe infections requiring prolonged antibiotic therapy, such as spondylodiscitis. Notably, TDM data showed that more than 90% of dalbavancin plasma concentrations exceeded the PK/PD target against staphylococci. In a separate study, Gatti et al. [38] investigated the association between the prolonged maintenance of conservative PK/PD targets for dalbavancin and clinical outcomes in 17 patients undergoing long-term therapy for staphylococcal osteoarticular infections. Patients received two 1500-mg doses administered one week apart. In most cases, dalbavancin PK/PD targets were maintained for the majority of the treatment period, resulting in favorable clinical outcomes. Similar results were subsequently reported by the same authors in patients with cardiovascular prosthetic infections [39]. More recently, Cojutti et al. described the implementation of a hub-and-spoke model aimed at optimizing long-term dalbavancin therapy for chronic staphylococcal infections through TDM-guided expert clinical pharmacological advice (ECPA) [40]. Dalbavancin was administered either with curative intent (curative group) or for suppressive purposes (suppressive group). Across 12 spoke hospitals, a total of 414 TDM-guided ECPAs were requested for 101 patients, with 64% receiving therapy for curative and 36% for suppressive indications. TDM-guided ECPAs optimized dalbavancin treatment for up to 14 months in the curative group and up to 28 months in the suppressive group, achieving median optimal exposures of 96% and 100%, respectively. In the curative group, maintaining dalbavancin concentrations above the optimal target for less than 70% of the treatment period was linked to a higher risk of therapeutic failure [odds ratio (OR) 6.71; 95% confidence interval (CI) 0.97–43.3; *p* = 0.05]. In the suppressive group, the presence of infective endocarditis was independently associated with an increased likelihood of treatment failure (OR 8.65; CI 1.29–57.62; *p* = 0.046).

Importantly, two real-world pharmacokinetic studies have provided important insights into the long-term exposure and clinical effectiveness of dalbavancin administered as either a single or two 1500 mg doses in outpatients with ABSSSIs and osteoarticular infections [41,42]. Overall, dalbavancin demonstrated prolonged plasma exposure, with T > MIC and AUC/MIC values largely exceeding previously proposed pharmacodynamic targets, particularly in the osteoarticular setting. Clinical success was achieved in the majority of patients and treatment was well tolerated. Notably, therapeutic failure was associated with lower pharmacokinetic exposure, especially reduced Cmax, which emerged as a potential predictor of outcome and a candidate parameter for TDM. Furthermore, the dual-dose regimen administered one week apart appeared to provide more sustained exposure and was suggested as the preferred strategy for osteoarticular infections.

Among other research groups, Lafon-Desmurs et al. reported their experience with a suppressive antibiotic therapy strategy based on dalbavancin TDM in 15 patients with implant-related infections [43]. The median number of dalbavancin injections was 4 (interquartile range: 2–7), and the median interval between reinjections was 57 days (IQR, 28–82). Dalbavancin plasma concentrations remained above 8 mg/L for 85% of the treatment duration.

Conversely, Soderquist et al. aimed to determine dalbavancin Cmin during long-term treatment in 12 patients with prosthetic joint infections, according to the Swedish National Guidelines for Bone and Joint Infections (loading dose of 1500 mg on day 1 and a second 1500 mg dose on days 8–14, followed by 1000 mg every two weeks or 500 mg weekly from day 28 onward) [44]. The median serum concentration 14 days after the first 1500-mg dose was 36 mg/L (range 7–62 mg/L). The median trough concentration at the time of the last administered 1000-mg dose, after a total of six to seven doses, was 54 mg/L (range, 32–98 mg/L). Three patients showed a tendency toward progressive dalbavancin accumulation during therapy.

## 8. The Proactive TDM of Dalbavancin

Proactive TDM involves the regular measurement of drug concentrations in a patient’s blood or plasma to guide preemptive dose adjustments, ensuring that drug levels remain within the desired therapeutic range. This approach contrasts with reactive TDM, where measurements are performed only after clinical concerns arise.

The application of proactive TDM for dalbavancin, based on the assessment of both Cmin and Cmax concentrations, was initially described by Cattaneo et al. [45,46]. These measurements are used to predict the timing of subsequent dalbavancin doses through individualized log-linear regression models, with the aim of maintaining Cmin levels ≥8 mg/L [29,45,46]. In their first study, 366 TDM assessments from 81 patients were collected between December 2021 and November 2023 [45]. Dalbavancin Cmin values ranged from 4 to 71 mg/L, while Cmax levels ranged from 75 to 996 mg/L. Using log-linear regression, the authors estimated that injections should be spaced approximately every 42–48 days to sustain Cmin above 8 mg/L. Notably, patients monitored using the Cmax-based approach received significantly fewer injections (5 ± 2 versus 7 ± 3; *p* = 0.005) over a longer median interval (40 ± 10 versus 29 ± 14 days; *p* = 0.013) compared with the Cmin-based group. Based on these findings, the Cmax-guided strategy was adopted to optimize the efficiency and precision of dalbavancin administration in patients requiring prolonged therapy.

Subsequently, the same group applied this prospective methodology to 16 adults with chronic osteoarticular infections receiving a median of seven dalbavancin injections (up to 12 doses over 15 months) [46]. Timing of administration was tailored individually to achieve optimal drug exposure, with injections spaced every 39–47 days on average, although in some cases intervals extended up to 69 days. This individualized approach has also proven effective across a wide spectrum of body mass indices (BMI <13 to >30 kg/m^2^) [20,28]. Across these studies, only 10% of TDM assessments resulted in Cmin levels <8 mg/L, primarily during the first two months when individual models were still being refined.

The analyses also confirmed substantial inter-individual variability in optimal dosing intervals, ranging from less than 20 to more than 70 days, whereas intra-individual variability remained very low (<10%). This indicates that, once individualized dosing intervals are established, they remain stable over time, enabling scheduled dalbavancin administration even in the absence of ongoing TDM. Importantly, the models accounted for BMI effects: in overweight and obese patients, optimal dosing intervals were shortened by approximately 8 and 13 days, respectively, while patients with a BMI <20 kg/m^2^ required an extension of about 5 days to maintain Cmin concentrations above 8 mg/L. As extensively discussed throughout the article, all published TDM studies of dalbavancin have adopted as their PK target the maintenance of Cmin concentrations above 8 mg/L.

However, it is important to emphasize that while breakpoint values are useful for population-based PK/PD analyses, in the individual patient setting, the precise MIC of the infecting pathogen is far more clinically relevant. In this context, a further advancement in the optimization and personalization of dalbavancin therapy involves incorporating the actual MIC of the causative pathogen into PTA assessments, rather than relying solely on breakpoint-derived values. This approach is now feasible owing to the availability of modern diagnostic techniques that allow accurate MIC determination at the patient level [47]. Consequently, patients infected by the same microbial species may exhibit different degrees of susceptibility to dalbavancin, thereby requiring individualized PK/PD targets.

**Figure 2:**
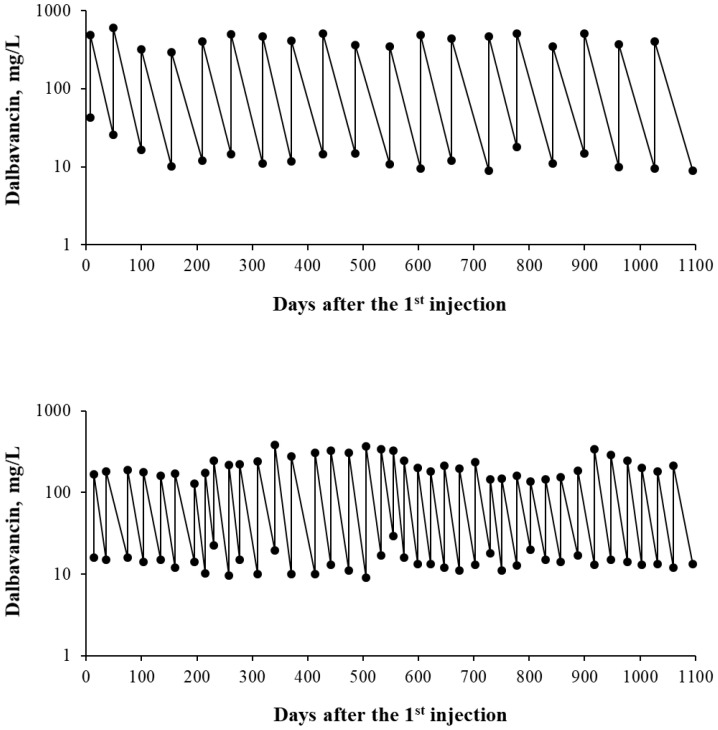
Time course of dalbavancin Cmax and Cmin concentrations over 3 years in two hypothetical patients treated with 1500 mg per injection, differing in renal function, BMI, and PK/PD targets (see text for details).

Based on the available literature, renal function, BMI, and MIC breakpoints are the primary factors influencing the timing of dalbavancin administration in patients requiring long-term antibiotic therapy. Figure 2 illustrates two hypothetical patients: the first (upper panel) is a woman with mild renal impairment (eGFR 65 mL/min) and a BMI of 21 kg/m^2^, receiving dalbavancin for a streptococcal infection with a Cmin target >8 mg/L; the second (lower panel) is a young, obese men (BMI 33 kg/m^2^) with normal renal function (eGFR 110 mL/min), receiving dalbavancin for a MRSA infection with a Cmin target >8 mg/L. Over a three-year treatment period, the first patient would receive 20 dalbavancin doses administered every 58 days, whereas the second patient would require 40 doses administered every 27 days. This hypothetical scenario demonstrates how TDM-guided scheduling of dalbavancin injections can result in markedly different dosing frequencies, with potential, but still largely unexplored, economic implications.

## 9. The Evolving Strategies for the Optimization of Dalbavancin Use

In this narrative review, we described the experience with traditional TDM, based on fixed plasma sampling (at a time corresponding to Cmin and Cmax). While this represents one approach to optimize and individualize dalbavancin administration timing, more advanced strategies are emerging to better predict drug exposure. In this context, in 2024, Cojutti et al. developed and validated a Bayesian approach to reliably predict the duration over which dalbavancin maintains optimal pharmacodynamic target attainment during long-term treatment of subacute and chronic staphylococcal infections [48]. The model was based on a two-compartment population pharmacokinetic framework constructed from data collected in 69 patients receiving dalbavancin for various subacute and chronic staphylococcal infections. Using this model, individualized dalbavancin PK profiles were generated for each patient through two approaches: an a priori approach, based solely on demographic and dosing data, and a posteriori or Bayesian approach, which incorporated measured drug concentrations. The study demonstrated that forecasting accuracy improved substantially with a posteriori approach, particularly when two randomly measured concentrations were available [48].

Subsequently, an in silico PK/PD simulation was conducted to evaluate the predicted dalbavancin concentrations resulting from suppressive regimens of 1000 mg or 1500 mg administered monthly, with or without a loading dose given one week after the initial administration [49]. The simulations indicated that free serum drug concentrations exceeded the PK target, defined as fAUC24/MIC > 27, in all patients receiving 1500 mg monthly, suggesting that an initial weekly loading dose may be unnecessary. However, the main limitation of this study lies in the selection of a relatively permissive PK/PD target (fAUC24/MIC > 27), which is approximately fourfold lower than the target adopted in previous studies (fAUC24/MIC > 111).

More recently, Sayadi et al. reported the development and validation of machine learning models designed to support individualized dalbavancin dosing in patients with complex infections [50]. These models integrate pre-second-dose plasma concentrations, patient-specific factors (such as age, body weight, and creatinine clearance), and pathogen MIC to optimize the timing of a third dose while minimizing the risk of sub-therapeutic exposure, thereby facilitating timely and effective treatment decisions. Support vector machine models demonstrated high performance, with accuracy exceeding 88% and sensitivity above 90% in both testing datasets and clinical validation cohorts. In clinical datasets, no false negatives were observed (noting the limited sample size), with overall predictive accuracy approaching 95%. Compared with maximum a posteriori Bayesian estimation, the machine learning approach achieved higher accuracy and sensitivity, particularly by reducing false-negative predictions, and reliably forecasted drug exposure through week 8, the relevant clinical period. This strategy allows early, individualized dosing adjustments using minimal patient data, complementing Bayesian approaches, reducing the need for repeated sampling, and providing a practical framework for model-informed precision dosing of dalbavancin.

## 10. Conclusions

A growing body of evidence consistently supports the role of proactive TDM in optimizing dalbavancin therapy for the management of complex and chronic infections. By enabling individualized adjustment of dosing intervals and ensuring sustained, adequate plasma concentrations over prolonged treatment courses, TDM enhances the probability of achieving PK/PD targets and may ultimately translate into improved clinical outcomes. This strategy could be further strengthened through a more refined and personalized approach to dalbavancin optimization, incorporating the actual MIC of the causative pathogen into PTA assessments, rather than relying exclusively on standardized breakpoint-derived values. Such an approach is now increasingly feasible due to the availability of advanced diagnostic methodologies that allow precise, patient-specific MIC determination, thereby enabling a truly individualized PK/PD-driven therapeutic strategy. The precision of this strategy can be further enhanced through the use of Bayesian forecasting, machine learning models, and streamlined model-informed precision dosing approaches. However, some important limitations should be acknowledged, including the lack of randomized controlled trials, the predominance of single-group research, the cost and accessibility of TDM, and the real-world feasibility of MIC-guided dosing strategies. Therefore, prospective multicenter studies are required to validate these methodologies and to establish well-defined, pathogen-specific PK/PD targets that can guide TDM-based dosing strategies across diverse patient populations.

## Figures and Tables

**Table 1:** Chromatographic methods for the quantification of dalbavancin in human blood samples.

Reference	Technology	Biological Matrix	Linearity	LOQ, mg/L	Sampling Feasibility
[30]	LC-MS/MS	Plasma/urine	0.5–500 mg/L	0.5	Cmin/Cmax
[31]	HPLC-UV	Serum	12.5–400 mg/L	12.5	Cmax
[32]	LC-MS/MS	Serum	1–50 mg/L	1.0	Cmin
[33]	ITD LC-MS/MS	Plasma	0.125–500 mg/L	0.125	Cmin/Cmax
[34]	HPLC-DAD	Plasma	5–100 mg/L	5	Cmin
[35]	LC-MS/MS	Plasma	6.25–200 mg/L	6.25	Cmin/Cmax
[36]	LC-MS/MS	Plasma	0.66–400 mg/L	0.66	Cmin/Cmax

ITD LC-MS/MS: Liquid Chromatography-Isotope Dilution Tandem Mass Spectrometry; HPLC-UV: high-performance liquid chromatography tandem ultraviolet detector; LC-MS/MS: liquid chromatography tandem mass spectrometry; DAD: diode array detector.

## Data Availability

No new data were created or analyzed in this study. Data sharing is not applicable to this article.

## Abbreviations

The following abbreviations are used in this manuscript:

ABSSSI	Acute bacterial skin and skin-structure infections
AUC	Area under the curve
BMI	Body mass index
CI	Confidence interval
CLCR	Creatinine clearance
Cmin	Minimum drug concentration
Cmax	Maximum drug concentration
DDI	Drug-drug interaction
ECPA	Expert clinical pharmacological advice
eGFR	Estimated glomerular filtration rate
IQR	Interquartile range
LOQ	Lower limit of quantification
MIC	Minimum inhibitory concentration
MRSA	Methicillin-resistant staphylococcus aureus
OR	Odds ratio
PD	Pharmacodynamics
PK	Pharmacokinetics
PTA	Probability of target attainment
TDM	Therapeutic Drug Monitoring
